# ndmaSNF: cancer subtype discovery based on integrative framework assisted by network diffusion model

**DOI:** 10.18632/oncotarget.21643

**Published:** 2017-10-06

**Authors:** Chao Yang, Shu-Guang Ge, Chun-Hou Zheng

**Affiliations:** ^1^ College of Computer Science and Technology, Anhui University, Hefei, Anhui 230601, China; ^2^ College of Electrical Engineering and Automation, Anhui University, Hefei, Anhui 230601, China

**Keywords:** cancer subtyping, integrative method, network diffusion, somatic mutation data

## Abstract

Recently, with the rapid progress of high-throughput sequencing technology, diverse genomic data are easy to be obtained. To effectively exploit the value of those data, integrative methods are urgently needed. In this paper, based on SNF (Similarity Network Diffusion) [1], we proposed a new integrative method named ndmaSNF (network diffusion model assisted SNF), which can be used for cancer subtype discovery with the advantage of making use of somatic mutation data and other discrete data. Firstly, we incorporate network diffusion model on mutation data to make it smoothed and adaptive. Then, the mutation data along with other data types are utilized in the SNF framework by constructing patient-by-patient similarity networks for each data type. Finally, a fused patient network containing all the information from different input data types is obtained by using a nonlinear iterative method. The fused network can be used for cancer subtype discovery through the clustering algorithm. Experimental results on four cancer datasets showed that our ndmaSNF method can find subtypes with significant differences in the survival profile and other clinical features.

## INTRODUCTION

Cancer is believed to be a complicated and heterogeneous disease since that it is driven by different combinations of mutated genes rather than the individual gene, and those mutations vary among tumor samples. Great efforts have been made by several large-scale projects such as The Cancer Genome Atlas (TCGA) [2], International Cancer Genome Consortium (ICGC) [3], and Cancer Cell Line Encyclopedia (CCLE) [4], etc., which generated a sea of multiple genomic platform data. Therefore, integrative methods are urgently needed to simultaneously employ those molecular data for identification of tumor subsets with different clinical and biological meaning.

Until now, many successful researches on such integrative framework for cancer subtype identification have been published. For instance, Liu et al. [5] brought forward a method using regularized non-negative matrix factorization for gene expression analysis. Liu et al. [6] also came up with an approach for integrated analysis via block-constraint robust principal component analysis. Gu et al. [7, 8] came up with approaches which had made progress in classification and regression. Shen et al. [9] proposed a joint latent variable model named iCluster which can realize data integration and dimensionality reduction simultaneously. Clustering result can be obtained by applying a standard K-means algorithm on the joint latent variable. Though pioneering and effective, iCluster to a great extent relies on the step of feature preselection. Wang et al. [1] introduced a distinct integrative approach called SNF which contains a few steps. First, for each data type, a sample-by-sample similarity network is constructed using the Euclidean distance and a scaled exponential similarity kernel, then these similarity networks are fused into one single network by a nonlinear iterative method. At last, this fused network is clustered by spectral clustering to receive several tumor groups. In SNF, diverse data such as DNA methylation, mRNA expression and miRNA expression data were used for identification of meaningful cancer subtypes. However, those data types are with continuous value for which the Euclidean metric is suitable. Obviously, it turns out to be helpless with discrete profile such as somatic mutation data. Indeed, for discrete data they do propose to use chi-squared distance (Supplementary Note-Chi-squared distance) to calculate the similarity between the patients, nevertheless by which we cannot get a satisfactory result.

There are intrinsic differences between mutation data and other data types with quantitative value: (i) mutation data has binary value so it is not suitable for Euclidean measurement; (ii) high-dimensionality makes typical binary similarity measures hard to be used; (iii) its sparseness (fewer than 100 genes mutated in nearly ten thousand genes) makes it heterogeneous such that clinically identical patients rare to share more than a single mutation. So it makes traditional distance-based similarity measurement impossible to be used. Actually, somatic mutation data has important value since it provides information about relationships between genes and biochemical pathways and comprehensive insight into tumor progress [10]. To deal with this problem, Hofree et al. [11] brought forward a method named NBS (network-based stratification) which integrated somatic mutation data with gene networks using network diffusion model and performed clustering in a consensus clustering framework to make result robust. It shows that somatic mutation data is a promising source for cancer subtype identification. However, NBS did not use any other levels of information data such as epigenome, transcriptome, etc.

**Figure 1:**
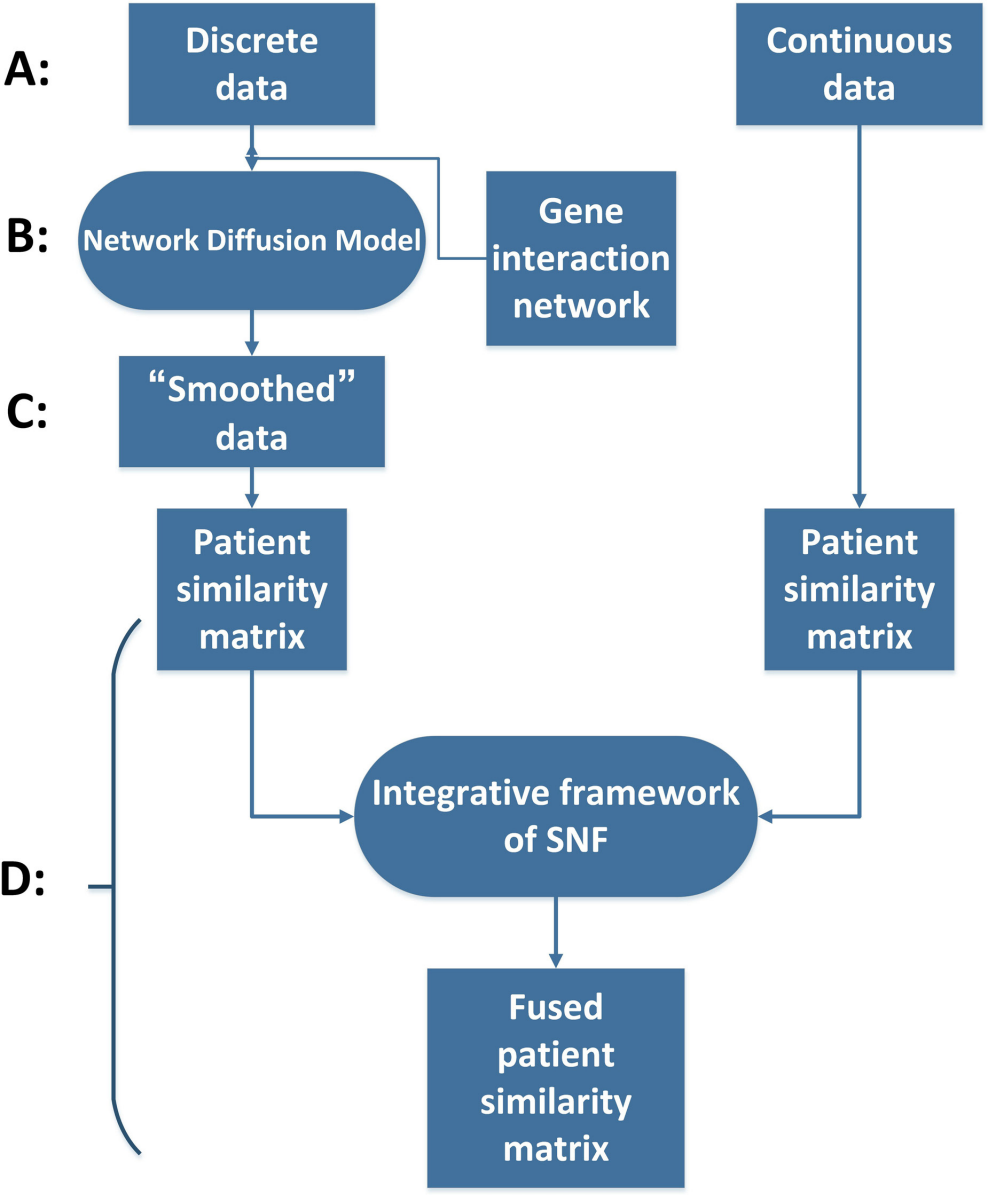
The flow chart of ndmaSNF **(A)** Dividing data into two main categories. **(B)** Pre-process for data types with discrete value via network diffusion model incorporating gene interaction network. **(C)** “Smoothed” mutation data. **(D)** All of these patient similarity matrices derived from various data types were combined into one fused patient similarity matrix through integrative framework of SNF.

In this paper, we proposed a method named ndmaSNF (network diffusion model assisted SNF) based on the integrative framework of SNF [1] for cancer subtype identifying using somatic mutation profile and other data from different platforms simultaneously. Figure 1 shows the schematic overview of our method. We roughly divided the data sources into two categories: continuous data and discrete data (Figure 1A). For discrete data (e.g. mutation status), we made it fit the framework of SNF by using network diffusion model (Figure 1B) along with gene interaction network. Then the discrete data was smoothed and could be used well via SNF framework together with those continuous data (Figure 1C). By combining similarity matrices from those two different kinds of data, a fused patient-by-patient similarity matrix was obtained through the nonlinear combination method used in SNF framework (Figure 1D). On this fused matrix, clustering result can be acquired by applying a clustering algorithm such as spectral clustering. We extensively applied ndmaSNF on several human cancer data sets consisted of various kinds of data types, and received biologically and clinically relevant cohorts of patients, with better *P* value and silhouette value compared to SNF. The clustering result broadly met the PAM50 classification indicated clinical value for treatment.

Moreover, existing methods generally identify network modules common to all tumors which may ignore the heterogeneity between various subtypes. In this study, we first use ndmaSNF on various data sources to gain cancer subtypes, and for each cancer subtype, we use DriverNet [12] to get potential driver genes. We then did pathway enrichment analysis on those genes per subtype. And the top 60 potential driver genes attained from DriverNet were used for subtype-specific network module discovery via software GenRev [13]. The experimental results indicated that our ndmaSNF has the ability to find distinct cancer subtypes relevant to different clinical outcomes and network modules.

## RESULTS

### Performance comparison

We evaluated the performance of our method ndmaSNF by comparing it with two state-of-the-art methods, i.e. SNF [1] and LRAcluster [14] via silhouette value and *P* value as metrics on four cancer datasets (BIC: breast invasive carcinoma; KRCCC: kidney renal clear cell carcinoma; LSCC: lung squamous cell carcinoma; COAD: colon adenocarcinoma). The experimental results are listed in Tables 1 and 2 (For *P* value, the lower the better; for silhouette value, the higher the better).

**Table 1:** Comparison of ndmaSNF with other methods on four cancer datasets using *P* value

	LSCC	KRCCC	BIC	COAD
SNF without mutation data	1.16E-03	8.76E-04	5.74E-06	3.38E-04
SNF	9.86E-04	1.45E-03	1.59E-06	3.56E-04
LRAcluster	4.30E-02	3.24E-02	5.70E-02	9.90E-03
ndmaSNF	2.83E-04	3.43E-04	2.46E-08	1.40E-04

**Table 2:** Comparison of ndmaSNF with other methods on four cancer datasets using silhouette value

	LSCC	KRCCC	BIC	COAD
SNF without mutation data	0.46	0.34	0.43	0.50
SNF	0.46	0.33	0.34	0.51
LRAcluster	0.50	0.32	0.46	0.35
ndmaSNF	0.52	0.39	0.45	0.43

In Table 1, the terms in second row (SNF without mutation data) mean that we used 3 continuous data types (DNA methylation, mRNA expression, miRNA expression). And the terms in other rows (SNF, LRAcluster and ndmaSNF) are the results with 4 data types including mutation data. By comparing the second row (SNF without mutation data) and the fifth row (ndmaSNF), we can see that somatic mutation profile is a promising data source for identification of cancer subtypes. However, the promising value of the mutation data was not reflected by using original SNF as the third row (SNF) shows. By comparing the second row and the third row, we can see that SNF didn’t exploit the mutation data well, and even may had a bad influence compared with result without mutation data (KRCCC, COAD). LRAcluster [14] is another integrative method with fast properties to find the shared principal subspace across multiple data types. However, it even didn’t perform well compared with original SNF. Due to its fastness, we think that LRAcluster has an advantage in large-scale data analysis such as pan-cancer analysis instead.

In terms of silhouette value, the promotion of our method compared with other methods was slight (Table 2). And for COAD cancer data set, performance of our method even decreased slightly, we attributed the result to the fact that COAD has at least one subtype with few patients, it makes the silhouette value very sensitive and unstable. However, we can at least conclude that the involvement of the mutation data did not destroy the combination of the original 3 data types (DNA methylation, mRNA expression, miRNA expression) used in SNF [1].

### A case study: breast invasive carcinoma

To further validate that our ndmaSNF can identify subtypes with biological and clinical differences, we then did in-depth research on breast invasive carcinoma. Breast invasive carcinoma (BIC) is a common breast cancer, growing into normal and healthy tissues.

**Figure 2:**
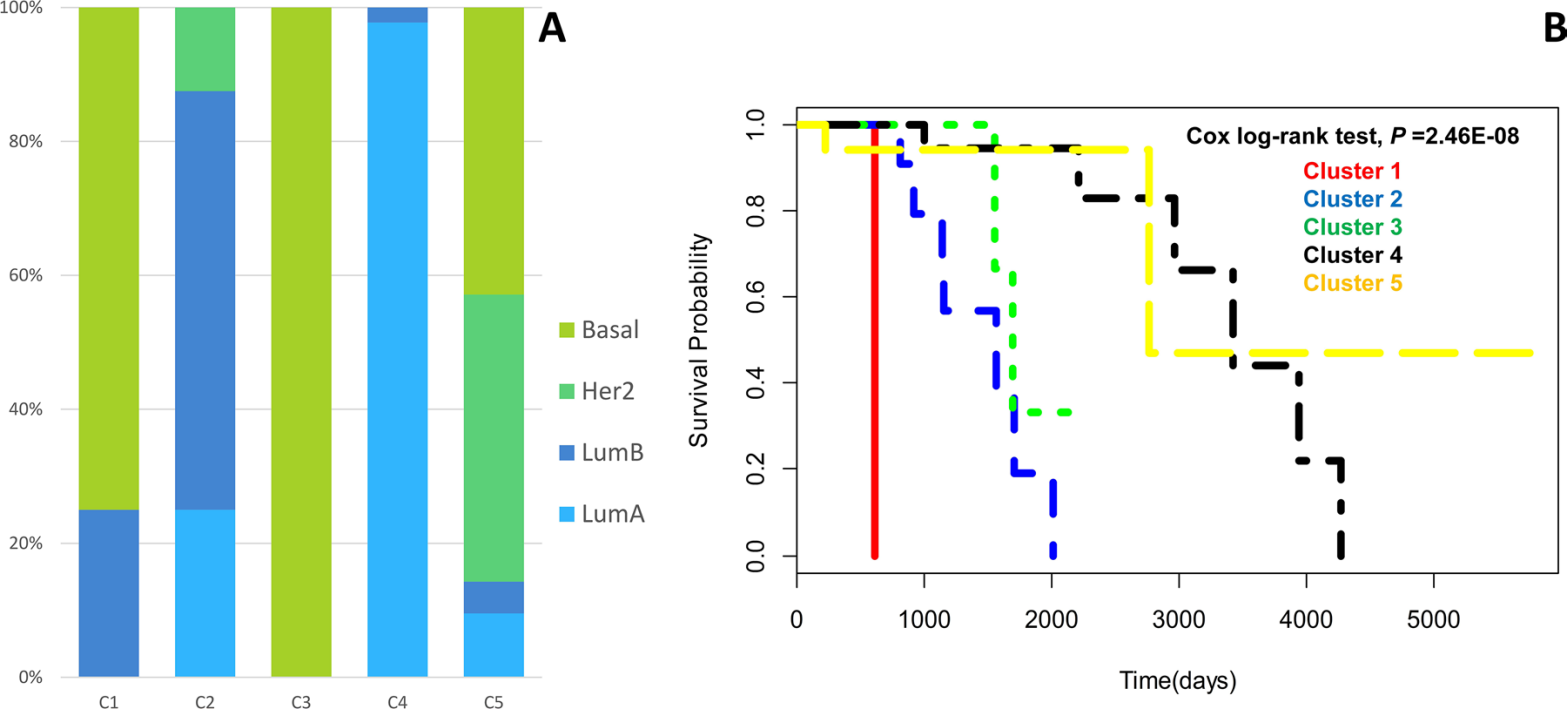
**(A)** Distribution of PAM50 samples in the identified subtypes. **(B)** Kaplan-Meier survival curves of 5 subtypes identified.

We totally identified 5 subtypes of BIC with log-rank *P* value of 2.46E-08. To show the extent of those subtypes discovered by our method corresponded to the established PAM50 classification, we gathered statistics of the distribution of PAM50 per subtype. C1-C5 in Figure 2A represents subtypes identified by our method. We can see that C3 and C4 are considerably fit the result of PAM50 classification: Basal-like for C3 and Luminal A for C4. And C1 is mostly consisted of Basal-like cases, C2 is mostly composed of luminal B cases. C5 is mostly comprised by Basal-like cases and Her2-enriched cases. Luminal A subtype is more likely to have a good prognosis while Basal-like subtype is aggressive and have a poorer prognosis, this can be reflected in Kaplan-Meier plot which shows an obvious significant survival difference in Figure 2B. C3 has significantly shorter overall survival durations than those with C4. Although C1 and C3 are both Basal-like subtypes, they have a difference in survival probability (*P* = 0.036) which can be seen in Figure 2A. C1 is more aggressive than C3 as the survival curves shows.

**Figure 3:**
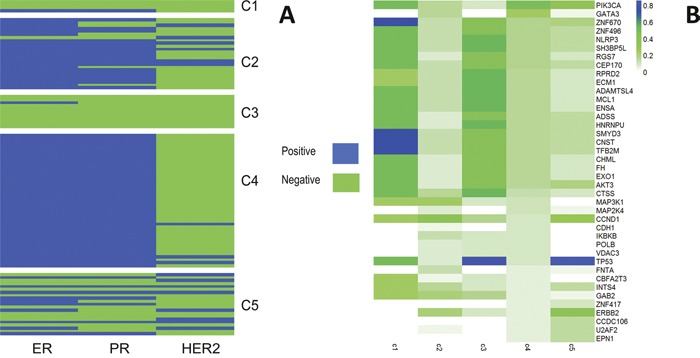
**(A)** Clinical features with ER/PR/HER2 per subtype. **(B)** Comparison of the mutation frequencies among the identified subtypes.

In Figure 3A, we can see that C1 and C3 are mainly triple negative while C2 and C4 are largely ER positive, PR positive and HER2 negative, however, the situation of C5 is somewhat complicated. Basal-like subtype breast cancer is usually triple negative, this verified the fact that C1 and C3 are mostly consisted of Basal-like cases in Figure 2A.

Furthermore, we turned to mutation frequencies for more validation. Thus, we focus on the genes with high mutation frequency and can find evident differences between each cluster (Figure 3B): *ZNF670, SNMYD3, CNST*, and *TFB2M* for cluster 1; *CCND1, MAP3K1* and *ERBB2* for cluster2; *TP53, CTSS, NLRP3, SH3BP5L* for cluster 3; *PIK3CA, GATA3* for cluster 4 and *TP53, PIK3CA, ERBB2* and *CCND1* for cluster 5. It shows that each subtype identified has a different combination of genes highly mutated and corresponded to various biological processes.

### Driver gene analysis per subtype identified in breast invasive carcinoma

To further study by what gene combination each subtype is driven, and whether those driver genes combination are different corresponded to different biological pathway, we applied DriverNet [12] to find important genes by using gene expression data, mutation data and gene-by-gene network.

In table 3, Note that *TP53* showed great importance in all subtypes, however, a total combination of top driver genes is distinct in each subtype. To clearly show the difference, we used the top 60 driver genes identified from each subtype to do further study including pathway enrichment analysis and network module identification. The aim is to find out what biological process and important pathway those driver genes from different subtypes participated in.

**Table 3:** Top 10 driver gene per subtype attained by DriverNet [5]

C1	C2	C3	C4	C5
TP53	TP53	TP53	TP53	TP53
CSNK2A1	MYC	MYC	PIK3CA	ERBB2
EP300	CCND1	CDKN2A	MYC	MYC
PRKCA	PAK1	RB1	IGF1R	PIK3R1
UBQLN4	CSNK2A1	STAT5A	MAP2K4	SMAD3
SHC1	ERBB2	MCL1	LRP2	ACTL6A
MYC	IGF1R	IGF1R	GATA3	TTN
CCDC85B	MAPT	TUBG1	MCL1	U2AF2
RELA	RELA	IKBKB	CDH1	SMAD2
PAK1	PIK3R1	BRCA1	TTN	CDKN2A

**Figure 4:**
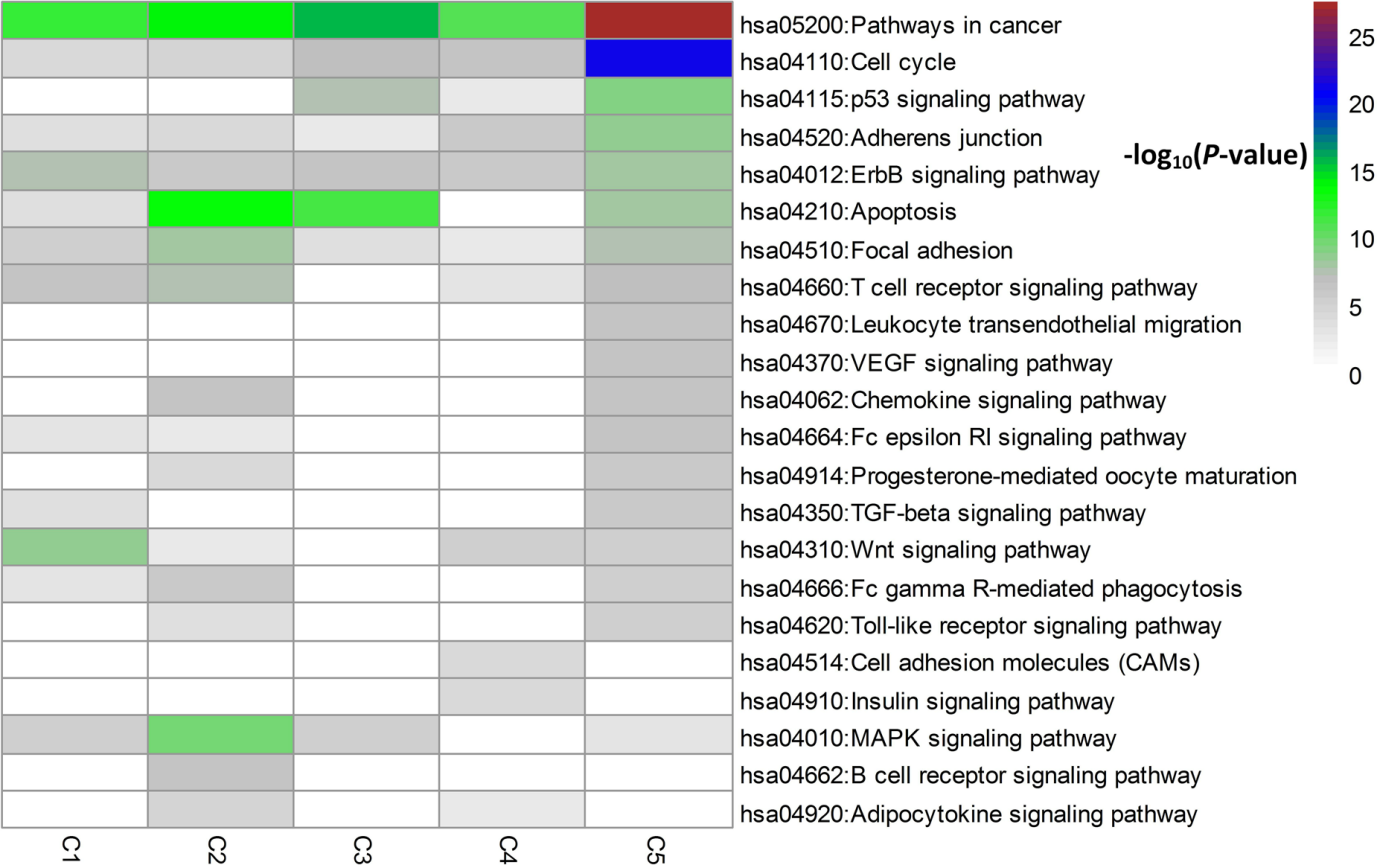
Pathway enrichment analysis for the top 60 driver genes per subtype

We did a KEGG pathway enrichment analysis per subtype and selected pathways related to breast cancer. From Figure 4, we can see differences between subtypes at the enrichment level. It is not surprising to see that all subtypes have an apparent enrichment in hsa05200: Pathways in cancer. Also, Apoptosis, a programmed cell death mechanism, is commonly enriched in C2, C3 and C5.

C1 is typically enriched in Wnt and ErbB signaling pathway. The Wnt signaling pathway is one of a group of signal transduction pathways made of proteins that pass signals into a cell through cell surface receptors. Wnt signaling is identified for its role in carcinogenesis. This pathway's clinical importance is demonstrated by mutations that lead to various diseases, including breast cancer [15]. Furthermore, excessive ErbB signaling is associated with the development of a wide variety of types of solid tumor [16].

C2 is typically enriched in MAPK signaling pathway. The MAPK is a chain of proteins in the cell that communicates a signal from a receptor on the surface of the cell to the DNA in the nucleus of the cell. When one of the proteins in the pathway is mutated, it can become stuck in the “on” or “off” position, which is a necessary step in the development of many cancers. Components of the MAPK/ERK pathway were discovered when they were found in cancer cells. Drugs that reverse the “on” or “off” switch are being investigated as cancer treatments [17].

C3 is typically enriched in p53 signaling pathway. In breast cancer, p53 mutation is associated with more aggressive disease and worse overall survival. Molecular pathological analysis of the structure and expression of constituents of the p53 pathway is likely to have value in diagnosis, in prognostic assessment and in treatment of breast cancer [18].

C4 is typically enriched in Cell cycle and Adherens junction. The cell cycle is the series of events that takes place in a cell leading to its division and duplication. Regulation of the cell cycle involves processes crucial to the survival of a cell, including the detection and repair of genetic damage as well as the prevention of uncontrolled cell division. Adherens junctions, the most common type of intercellular adhesions, are important for maintaining tissue architecture and cell polarity and can limit cell movement and proliferation.

C5 is typically enriched in many pathways represented in C1-C4 such as Cell cycle, p53 signaling pathway, Adherens junction, ErbB signaling pathway and Apoptosis. This also can be reflected in Figure 2A: C5 is a mixture of different PAM50 subtypes.

### Network module identification and analysis per subtype identified in breast invasive carcinoma

To get a more clear understanding of the combination of different driver genes, we seek for their significance at network module level. Therefore, we used them as seed genes to find network module per subtype.

**Figure 5:**
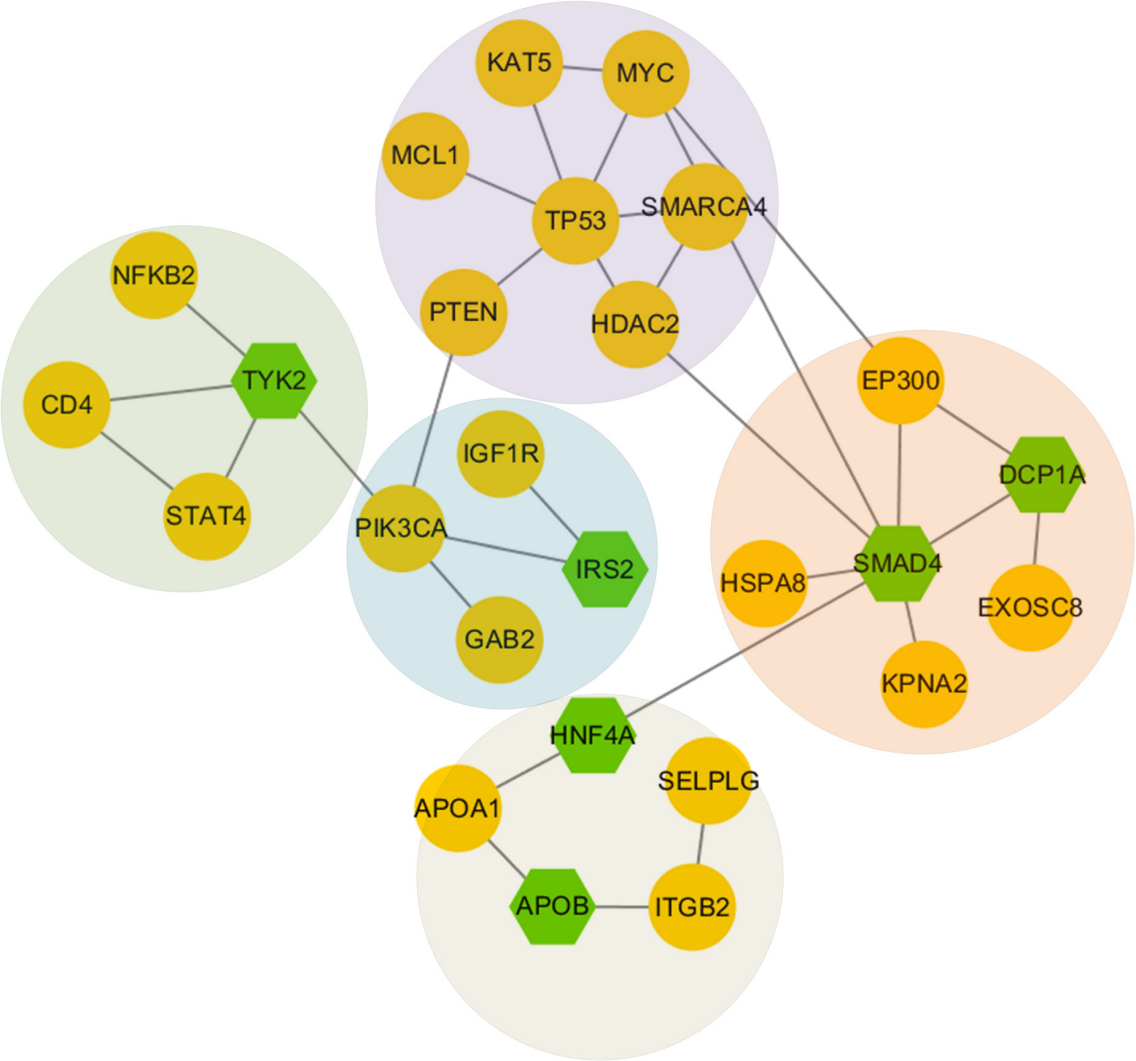
Network modules discovered in subtype 1 The green nodes represent genes we input, and the yellow nodes represent linker genes connecting those genes we input.

For subtype C1, we used top 60 driver genes as seed genes and 42 genes connected to each other on gene interaction network by utilizing GenRev [13]. We totally found 10 network modules and 5 of which are connected to each other and have more than 4 genes with the division modularity of 0.53. Those 5 modules comprised the largest sub-network (Figure 5). The densest module is TP53 module which contains many important genes related to breast cancer. *TP53* is a well-known tumor suppressor gene associated with various cancers including breast invasive carcinoma. Its mutation status and gene-expression based groups are important survival markers of breast cancer, and these molecular markers may provide prognostic information that complements clinical variables [19]. TP53 module also contained *SMARCA4*, which can inhibit the cells’ ability to migrate and invade. So it attaches an importance to pathogenesis of breast cancer as a prognostic marker together with a possibly selective therapeutic target [20]. *HDAC2* is another important gene related to breast cancer that is inclined to strongly express in aggressive breast cancer tumor subgroups [21]. We also discovered a SMAD4 module. Current research shows that *SMAD4* plays a key role in both tumor suppression and progression of breast cancer cells [22]. Another critical gene included in this module is *EP300*, it encodes the transcriptional cofactor p300, which is highly expressed in diverse human cancers. Specially, the over expression of p300 in breast cancer predicts tumor recurrence and adverse prognosis [23]. The remaining three modules contain some other important genes such as *PIK3CA, TYK2* and *APOA1*, respectively. *PIK3CA* is a well-known oncogene in human cancers. Accumulating evidence suggests that mutation of *PIK3CA* is an early event in breast cancer and is more likely to play a role in breast tumor initiation than in invasive progression [24]. The role of *TYK2* is confirmed by biological experiments in suppressing the growth and metastasis of breast cancer [25]. For *APOA1*, it is one of the most significant genes correlated with the proteomic profile that are closely related to breast cancer and may be involved in robust detection of disease progression [26].

**Figure 6:**
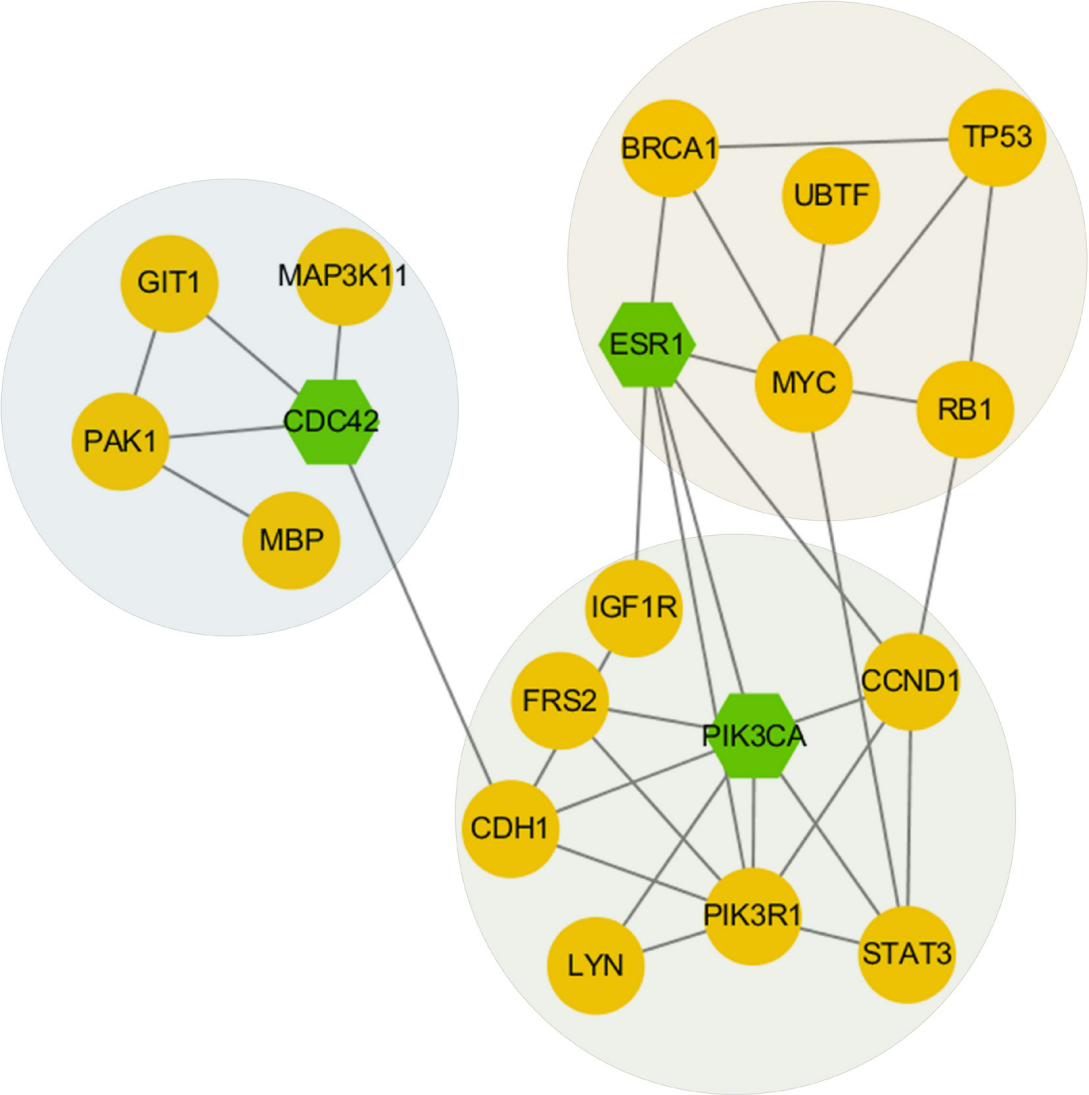
Network modules discovered in subtype 2

For subtype C2, after inputting top 60 driver genes as seeds, 41 genes were retained and we wholly got 8 modules with the division modularity of 0.50. The most densely connected sub-network is shown in Figure 6. The ESR1 module contained some important genes such as *ESR1*. Recent studies suggest that activating mutations in *ESR1* are a key mechanism in acquired endocrine resistance in breast cancer therapy [27]. The PIK3CA module contained some important genes such as *STAT3* and *PIK3R1*. Current findings show that activated *STAT3* signaling contributes to breast cancer progression and resistance to chemotherapy by inducing expression of the antiapoptotic protein, Survivin in part [28]. *PIK3CA* mutations and *PIK3R1* underexpression show opposite effects on patient outcome and could become useful prognostic and predictive factors in breast cancer [29]. We also identified a CDC42 module including important genes such as *CDC42* and *PAK1*. Growth and motility inhibition of breast cancer cells by epidermal growth factor receptor degradation is correlated with inactivation of *CDC42* [30]. And study shows associations between *PAK1* expression and subcellular localization in tumor cells and tamoxifen resistance [31].

We also did network module analysis for C3, C4 and C5, the results are given in Supplementary Materials (Supplementary Results-network module analysis for C3, C4 and C5).

## DISCUSSION

Integrative methods are urgently needed to exploit multiple genomic platform data simultaneously and get insight into human neoplasia, such as identification of cancer subtypes. In our work, we proposed a method named ndmaSNF by extending SNF, an integrative framework, to make full use of somatic mutation data. By using a network diffusion model, the somatic mutation data was “smoothed” and its value can be exploited to a large extent. The experimental results on several cancer data sets indicated that our method outperformed in identification of patients cohort with biological and clinical meaning. For example, we totally find 5 subtypes C1-C5 in BIC with different biological and clinical features. C3 is mostly consisted of Basal-like cases whereas C4 is mostly composed of luminal A cases. And the prognosis of C4 is better than C3. Interestingly, C5 is a mixture of different PAM50 subtypes and is typically enriched in many pathways represented in C1-C4. According to those subtypes, we did a deeper analysis including pathway enrichment analysis and network module identification. The results showed that our method could capture biological and clinical features effectively. Our research also demonstrated the value of the mutation data in giving insight into tumorigenesis. In the future, we will use some other discrete data such as copy number variations to make our method more compatible.

## MATERIALS AND METHODS

### Datasets

The data (DNA methylation, mRNA expression, miRNA expression) we used in this paper including four cancer data sets from TCGA website (https://cancergenome.nih.gov/), which have been processed and provided by Wang et al. [1]. And the mutation data of those four cancer data sets were obtained from UCSC data portal (http://genome.ucsc.edu/). We restricted our analysis to the 85 TCGA LSCC cases, 75 TCGA COAD cases, 101 TCGA KRCCC cases and 105 TCGA BIC cases, for which all DNA methylation, mRNA expression, miRNA expression and somatic mutation data were available. We used PPI (protein-protein interaction) network data obtained from NBS [11] after processing, with 11491 genes as gene interaction network.

### SNF integrative framework

Suppose we have n samples (*X*_1_, *X*_2_ … *X*_n_) which possess several data sources on multi-scale level (e.g. mutation data, expression data). We want to use these data simultaneously for identification of cancer subtype. The SNF framework can be described as follows.

First, for each data type, an nn patient similarity matrix *W* was constructed with its entry *W(i, j)* demonstrating the similarity between *X_i_* patient and patient *X_j_*. The specific formula to calculate *W* is as follows:

$$W\left(i,j\right)=\exp\left(-\frac{\rho^{2}\left(x_{i},x_{j}\right)}{\mu \varepsilon_{i,j}}\right)$$ (1)

Here $\rho \left(X_{i},X_{j}\right)$ represents the Euclidean distance between patient *X_i_* and patient *X_j_*. And *μ* is an empirical hyper parameter which is recommended to be set in the range of [0.3, 0.8]. Furthermore, *ε_i, j_* is defined as follows:

$$\varepsilon_{i,j}=\frac{mean\left(\rho \left(x_{i},N_{i}\right)\right)+mean\left(\rho \left(x_{j},N_{j}\right)\right)+\rho \left(x_{i},x_{j}\right)}{3}$$ (2)

Here $mean\left(\rho \left(X_{i},N_{i}\right)\right)$ is the average of the sum of the distances between *X_i_* and each of its neighbors. Obviously, the Euclidean distance measure is suitable for continuous variables. For discrete data, the chi-square distance is proposed (Supplementary Note-Chi-squared distance). There are two derivatives of matrix *W*, namely, matrix *P* and matrix *S*. Matrix *P* carries the full information about the similarity of each patient to all others obtained by performing normalization on *W*:

$$P\left(i,j\right)=\left\{ \begin{array}{l} \frac{W\left(i,j\right)}{2\sum_{k\neq i}W\left(i,k\right)},j\neq i\\ \;\;\;1/2,j=i \end{array}\right.$$ (3)

Matrix *S* only encodes the similarity to the *K* most similar patients for each patient via *K* nearest neighbors (KNN):

(4) $$S\left(i,j\right)=\left\{ \begin{array}{l} \frac{W\left(i,j\right)}{\sum_{k\in N_{i}}W\left(i,k\right)},j\in N_{i}\\ \;\;\;0,\mathrm{otherwise} \end{array}\right.$$

where *N_i_* represents a set of *X_i_*’s neighbors including *X_i_*. By using *P* as the global structure and *S* capturing local structure, a nonlinear iterative procedure is proposed:

$$P^{\left(v\right)}=S^{\left(v\right)}\times \left(\frac{\sum_{k\neq v}P^{\left(k\right)}}{m-1}\right)\times {\left(S^{\left(v\right)}\right)}^{T},v=1,2,...,m$$ (5)

where *P*^(*V*)^ represents *P* calculated from the *v*-th data profile. This procedure updates every *P*^(*V*)^ each time by *m* parallel interchanging diffusion processes. After *t* steps, the fused matrix *P*^(*C*)^ can be learned by taking average of all *P*^(*V*)^.

### Network diffusion model

We proposed to apply network diffusion model [32] incorporating gene interaction network on mutation profile and other discrete data. By using this method, the discrete data was “smoothed” and carries the information about similarity of tumor sample at the pathway level rather than the individual gene level, thus making SNF integrative framework work suitably and effectively on discrete data.

We first mapped patient mutation profile onto a gene interaction network. Then network diffusion model was applied to diffuse the effect of each mutated gene over this network for each patient according to the function:

$$F_{t+1}=\alpha F_{t}A+\left(1-\alpha \right)F_{0}$$ (6)

**Figure 7:**
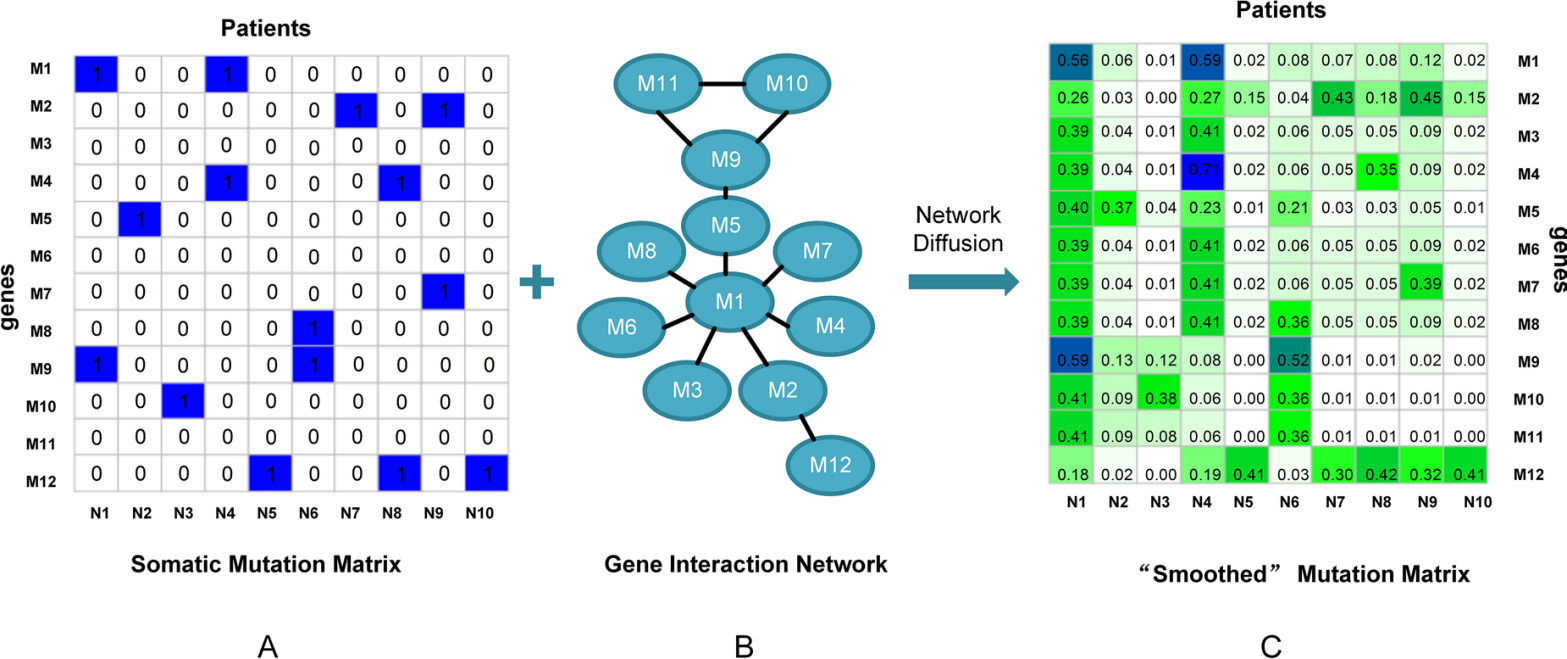
Simple presentation on network diffusion model **(A)** Somatic mutation data. **(B)** Gene interaction network. **(C)** “Smoothed” mutation data via network diffusion model.

*F*_0_is the binary patient-by-gene mutation data (Figure 7A), and *A* is a degree-normalized adjacency matrix of the gene interaction network (Figure 7B).α is used to adjust the distance that the mutation signal can propagate in the network. It is a tuning parameter in the optimal range of [0.5, 0.8]. The diffusion function run iteratively until *F*_*t*+1_ converges $\left(F_{t+1}-F_{t}<1\times 10^{-6}\right)$. The result *F*_*t*+1_ obtained is a “smoothed” mutation profile with quantitative value indicating the influence of each mutation per patient through network diffusion (Figure 7C). In this way, not only genes that are mutated will get high influence scores, but also genes that are close to the mutated genes in the network. According to this “smoothed” matrix, we seek for patient similarity as mutational consistency at pathway level rather than individual gene level. The benefit is 2-fold: (i) by “smoothing”, the sparseness is reduced, so the traditional distance measurement is feasible. (ii) in network diffusion model, mutation consistency is searched at pathway level rather than individual gene level, thus it will give a more comprehensive insight into similarity between patients. Since tumor process is driven by a combination of mutated genes, those genes’ influence is propagated through gene interaction network, so the tumor similarity at pathway level is more biologically significant and can improve the identification of cancer subtype.

### Evaluation metrics

To compare the performance of our method with established methods, we chose two metrics as evaluation index. First, we used *P* value for log-rank test of survival analysis by using survival time. *P* value measures the degree of significant difference between survival data of different subtypes. The lower the *P* value is, the more obvious the difference between subtypes is. For survival analysis, we took the same method used in SNF [1], thus we used the number of days to the last follow-up and vital status. However, for COAD, due to many missing values, these are combined with the number of days of last known living.

We also used silhouette value [33] to measure the quality of the clustering result. The silhouette value ranges from -1 to 1, where a high value indicates that the patient is well matched to its own cluster and poorly matched to other clusters. Then the mean value of silhouette value for all the patients was used as a measure of the compactness within clusters and the separation among clusters.

## SUPPLEMENTARY MATERIALS FIGURES


