# Supplementary material for: ndmaSNF: cancer subtype discovery based on integrative framework assisted by network diffusion model
Source: Oncotarget. 2017 Oct 6;8(51):89021–32. doi: 10.18632/oncotarget.21643 (PMC5687665; doi:10.18632/oncotarget.21643)
Supplement: Supplementary file 1 [file oncotarget-08-89021-s001.pdf]

ndmaSNF: cancer subtype discovery based on integrative framework assisted by network diffusion model

SUPPLEMENTARY MATERIALS

SUPPLEMENTARY NOTE

Chi-squared distance

Chi-squared distance is a measurement based on chi-square test. A chi-square test for independence compares two variables in a contingency table to see if they are related. The formula for the chi-square statistic used in the chi square test is:

$$\chi_c^2 = \sum \frac{(O_i - E_i)^2}{E_i}$$

Here the subscript “c” are the degrees of freedom. “O” is your observed value and E is your expected value.

DriverNet [1]

DriverNet is an integrated algorithmic framework for identification of pathogenic driver mutations by virtue of their effect on mRNA expression networks. DriverNet formulates associations between mutations and expression levels using a bipartite graph. The set of genes in the left partition of the graph represents mutation status and the set of genes right represents outlying expression status in each of patients. For each patient, an edge between the nodes on the right and right partitions of the graph is linked if the following three conditions are all satisfied: i) gene g_i is mutated in patient p of the population; ii) gene g_j shows outlying expression in patient p; iii) g_i and g_j are known to interact according to prior knowledge (e.g. pathway). Then a greedy optimization approach is applied to explain as many nodes on the right to explain as many nodes on the right partition of the bipartite graph as possible using the fewest number of nodes on the left partition of the graph such that the genes explaining the highest number of outlying expression events are nominated as putative driver genes. At last, statistical significance tests was applied to these candidates based on null distributions. See [1] for details.

GenRev [2]

GenRev is a network-based software package developed to explore the functional relevance of genes in molecular networks. By inputting biological network, gene list and gene scores (optional), the following two procedures are proposed: subnetwork extraction through three alternative algorithms (see [2] for details) and subnetwork analysis including several works such as visualization for network module, module detection, gene ranking and other follow-up analyses.

SUPPLEMENTARY RESULTS

network module analysis for C3, C4 and C5

For subtype3, we used top 60 driver genes as seed genes and 42 genes were retained by utilizing GenRev. We wholly got 10 modules with the division modularity of 0.52. The most densely connected sub-network is shown in Supplementary Figure 1.

The FANCA module contained some important genes such as FANCA. Sequence variants in FANCA could be potential spoilers of the Fanconi-BRCA pathway and as a result, they could in turn have an impact in non-BRCA1/2 breast cancer families [3].

We also identified a NCOR2 module. The associations of NCOR2 and CITED2 with outcome in oestrogen receptor-positive breast cancer patients underscore the clinical relevance of functional genetic screens to better understand disease progression, which may lead to the development of improved treatment options [4].

The STAT5A module contained some important genes such as STAT5A and ETS2. Low levels of Stat5a protein in breast cancer are associated with tumor progression and unfavorable clinical outcomes. Loss of STA5A represents a new independent marker of poor prognosis in node-negative breast cancer and may be a predictor of response to antiestrogen therapy if validated in randomized clinical trials [5]. Ets2 Maintains hTERT Gene Expression and Breast Cancer Cell Proliferation by Interacting with c-Myc*[6].

For subtype4, we used top 60 driver genes as seed genes and 40 genes were retained by utilizing GenRev. We wholly got 10 modules with the division modularity of 0.57. The most densely connected sub-network is shown in Supplementary Figure 2. The FYN module contained some important genes such as FYN. Recent study showed that FYN promotes breast cancer progression through

epithelial-mesenchymal transition [7]. We also identified a RPL5 module. In conclusion, RPL5 heterozygous inactivation occurs at high incidence in multiple tumor types, and a tumor suppressor role for RPL5 in breast cancer was identified [8]. The PRKACA module contained some important genes such as PRKACA and ERBB2. PRKACA mediates resistance to HER2-targeted therapy in breast cancer cells and restores anti-apoptotic signaling [9]. Overexpression of ERBB2, a receptor-like tyrosine kinase, is shared by several types of human carcinomas. Already, antibodies to ERBB2 are used in combination with chemotherapy in the treatment of metastasizing breast cancer [10].

For subtype5, we used top 60 driver genes as seed genes and 43 genes were retained by utilizing GenRev. We wholly got 14 modules with the division modularity of 0.57. The most densely connected sub-network is shown in Supplementary Figure 3. The CHEK2 module contained some important genes such as CHEK2 and TOPBP1. The results in [11] are consistent with the hypothesis that CHEK2 multiplies the risks associated with susceptibility alleles in other genes to increase the risk of breast cancer. TopBP1 is crucial for DNA damage and replication checkpoint controls. Based on its biological significance, it was inferred that TOPBP1 is a plausible susceptibility gene for hereditary breast and/or ovarian cancer [12]. The remaining modules contained some other important genes such as SKP2 and GAB2, respectively. It was concluded that SKP2 has oncogenic potential in breast epithelial cells and is overexpressed in a subset of breast carcinomas (ER- and Her-2 negative) for which SKP2 inhibitors may represent a valid therapeutic option [13]. GAB2 may be a key gene within an 11q13 amplicon in human breast cancer and propose a role for overexpression of GAB2 in mammary carcinogenesis. Agents that target GAB2 or GAB2-dependent pathways may be useful for treating breast tumors that overexpress GAB2 or HER2 or both [14].

1. Bashashati A, Haffari G, Ding J, Ha G, Lui K, Rosner J, Huntsman DG, Caldas C, Aparicio SA, Shah SP. DriverNet: uncovering the impact of somatic driver mutations on transcriptional networks in cancer. Genome Biol. 2012; 13:R124.
2. Zheng S, Zhao Z. GenRev: exploring functional relevance of genes in molecular networks. Genomics. 2012; 99:183-188.
3. Litim N, Labrie Y, Desjardins S, Ouellette G, Plourde K, Belleau P, Durocher F. Polymorphic variations in the FANCA gene in high-risk non-BRCA1/2 breast cancer individuals from the French Canadian population. Mol Oncol. 2013; 7:85-100.
4. Green AR, Burney C, Granger CJ, Paish EC, El-Sheikh S, Rakha EA, Powe DG, Macmillan RD, Ellis IO, Stylianou E. The prognostic significance of steroid receptor co-regulators in breast cancer: co-repressor NCOR2/SMRT is an independent indicator of poor outcome. Breast Cancer Res Treat. 2008; 110:427-437.
5. Peck AR, Witkiewicz AK, Liu C, Klimowicz AC, Stringer GA, Pequignot E, Freydin B, Yang N, Ertel A, Tran TH. Low levels of Stat5a protein in breast cancer are associated with tumor progression and unfavorable clinical outcomes. Breast Cancer Res. 2012; 14:R130.
6. Xu D, Dwyer J, Li H, Duan W, Liu JP. Ets2 maintains hTERT gene expression and breast cancer cell proliferation by interacting with c-Myc. J Biol Chem. 2008; 283:23567-23580.
7. Xie YG, Yu Y, Hou LK, Wang X, Zhang B, Cao XC. FYN promotes breast cancer progression through epithelial-mesenchymal transition. Oncol Rep. 2016; 36:1000.
8. Fancello L, Kampen KR, Hofman IJ, Verbeeck J, De Keersmaecker K. The ribosomal protein gene RPL5 is a haploinsufficient tumor suppressor in multiple cancer types. Oncotarget. 2017; 8:14462. https://doi.org/ 10.18632/oncotarget.14895.
9. Moody SE, Schinzel AC, Singh S, Izzo F, Strickland MR, Luo L, Thomas SR, Boehm JS, Kim SY, Wang ZC. PRKACA mediates resistance to HER2-targeted therapy in breast cancer cells and restores anti-apoptotic signaling. Oncogene. 2015; 34:2061-2071.
10. Harari D, Yarden Y. Molecular mechanisms underlying ErbB2/HER2 action in breast cancer. Oncogene. 2000; 19:6102.
11. Consortium CBCC-C. CHEK2* 1100delC and susceptibility to breast cancer: a collaborative analysis involving 10,860 breast cancer cases and 9,065 controls from 10 studies. Am J Hum Genet. 2004; 74:1175-1182.
12. Karppinen SM, Erkko H, Reini K, Pospiech H, Heikkinen K, Rapakko K, Syväoja JE, Winqvist R. Identification of a common polymorphism in the TopBP1 gene associated with hereditary susceptibility to breast and ovarian cancer. Eur J Cancer. 2006; 42:2647-2652.
13. Signoretti S, Di Marcotullio L, Richardson A, Ramaswamy S, Isaac B, Rue M, Monti F, Loda M, Pagano M. Oncogenic role of the ubiquitin ligase subunit Skp2 in human breast cancer. J Clin Invest. 2002; 110:633-641.
14. Bentires-Alj M, Gil SG, Chan R, Wang ZC, Wang Y, Imanaka N, Harris LN, Richardson A, Neel BG, Gu H. A role for the scaffolding adapter GAB2 in breast cancer. Nat Med. 2006; 12:114-121.

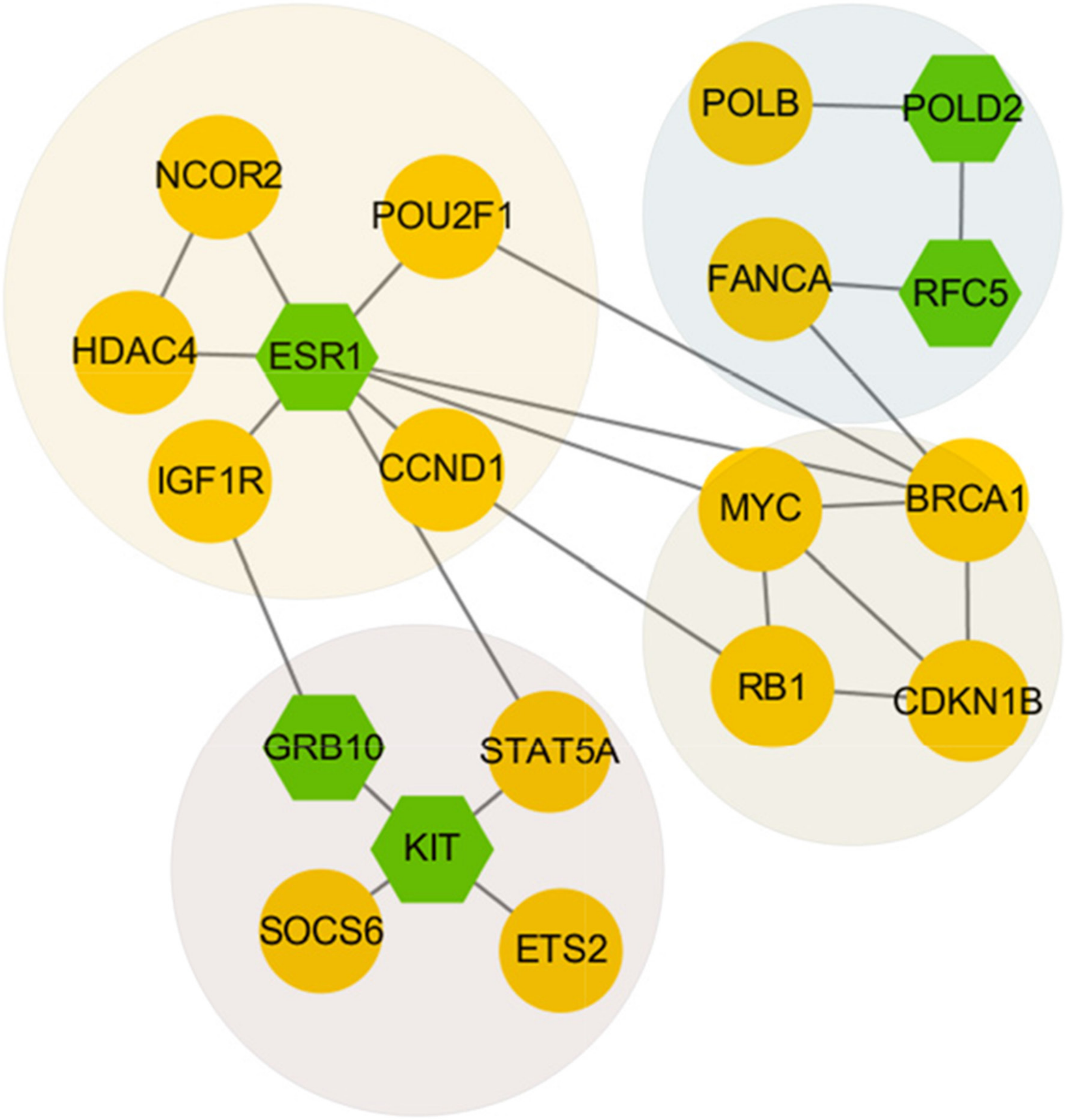

Supplementary Figure 1: Network modules discovered in subtype 3.

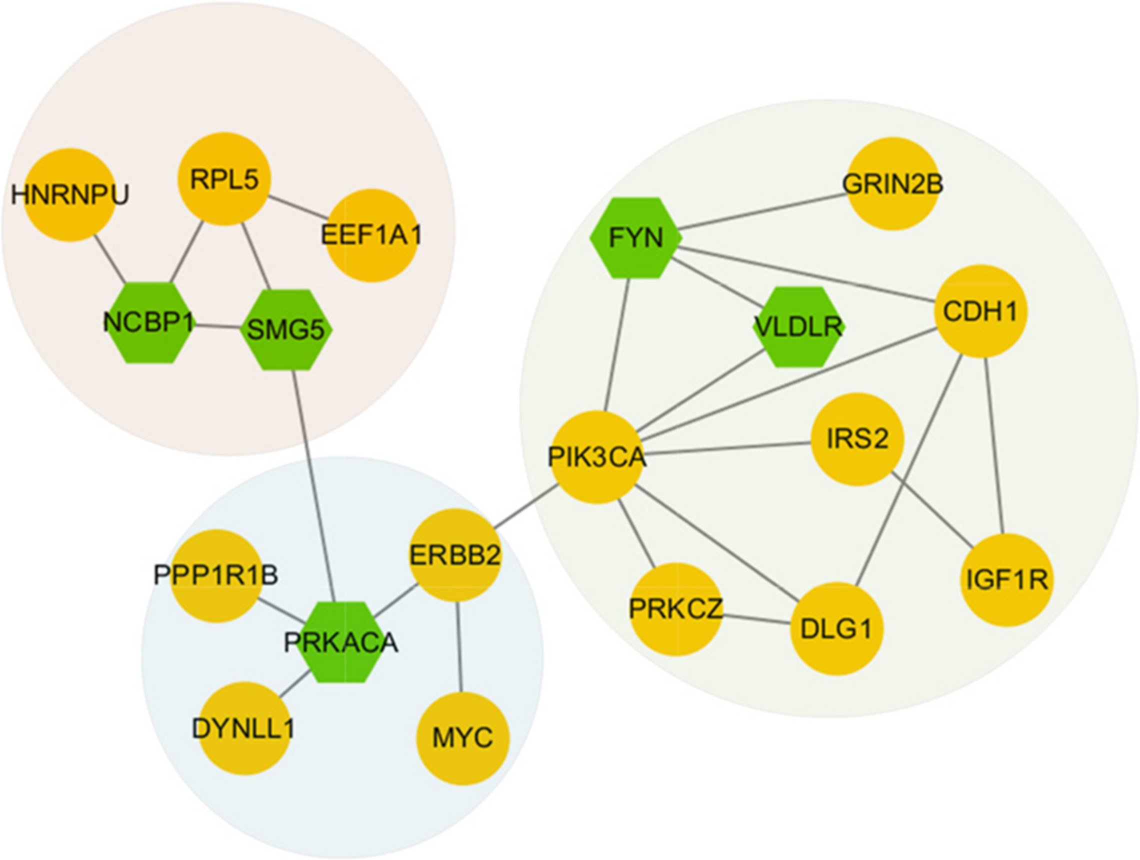

Supplementary Figure 2: Network modules discovered in subtype 4.

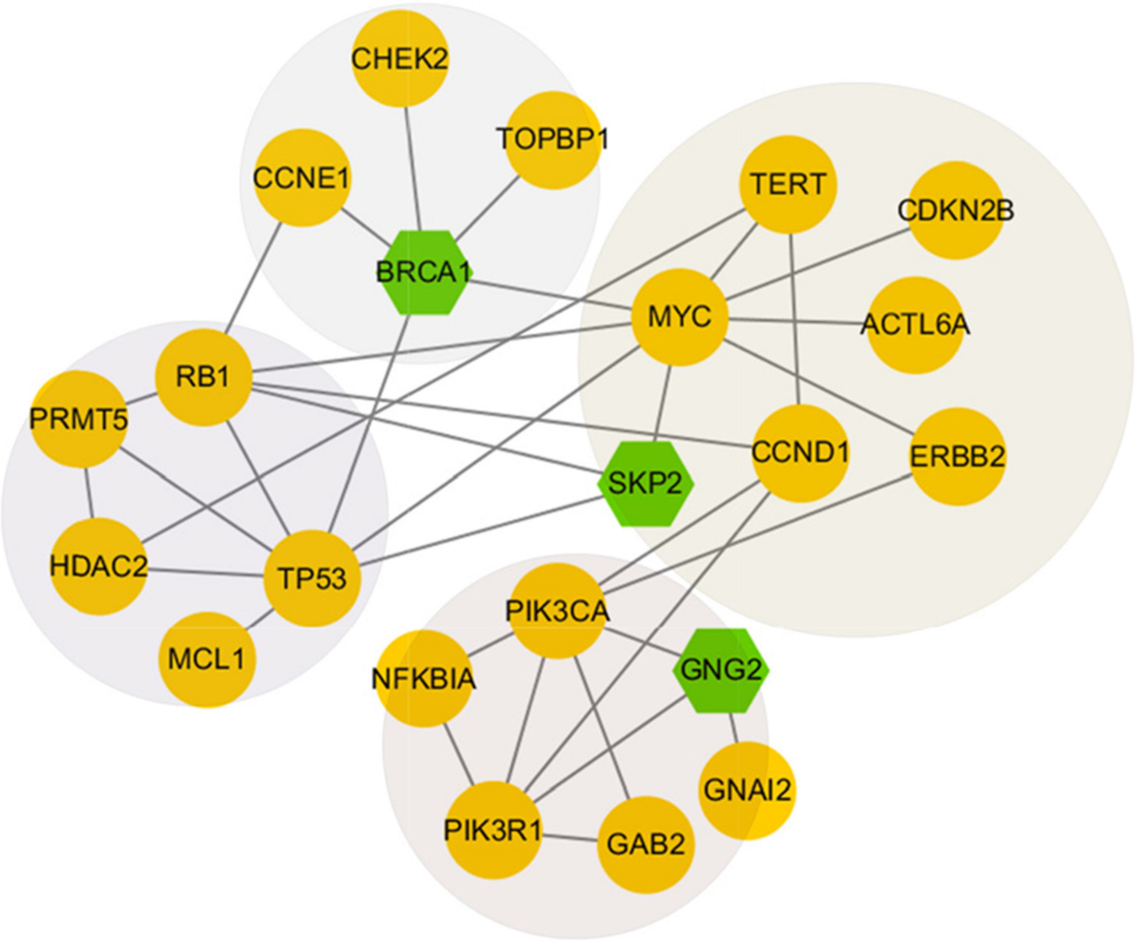

Supplementary Figure 3: Network modules discovered in subtype 5.
